# Survival and Long-Term Biochemical Cure in Medullary Thyroid Carcinoma in Denmark 1997–2014: A Nationwide Study

**DOI:** 10.1089/thy.2018.0564

**Published:** 2019-03-11

**Authors:** Jes Sloth Mathiesen, Jens Peter Kroustrup, Peter Vestergaard, Kirstine Stochholm, Per Løgstrup Poulsen, Åse Krogh Rasmussen, Ulla Feldt-Rasmussen, Sten Schytte, Stefano Christian Londero, Henrik Baymler Pedersen, Christoffer Holst Hahn, Jens Bentzen, Sören Möller, Mette Gaustadnes, Maria Rossing, Finn Cilius Nielsen, Kim Brixen, Anja Lisbeth Frederiksen, Christian Godballe

**Affiliations:** ^1^ Department of ORL—Head & Neck Surgery and Audiology, Odense University Hospital, Odense, Denmark.; ^2^ Department of Clinical Research, University of Southern Denmark, Odense, Denmark.; ^3^ Department of Clinical Medicine and Endocrinology, Aalborg University Hospital, Aalborg, Denmark.; ^4^ Steno Diabetes Center North Jutland, Gentofte, Denmark.; ^5^ Department of Internal Medicine and Endocrinology, Aalborg University Hospital, Aalborg, Denmark.; ^6^ Center for Rare Diseases, Aalborg University Hospital, Aalborg, Denmark.; ^7^ Department of Medical Endocrinology, Copenhagen University Hospital, Copenhagen, Denmark.; ^8^ Department of ORL—Head & Neck Surgery and Aarhus University Hospital, Aarhus, Denmark.; ^9^ Department of ORL—Head & Neck Surgery, Aalborg University Hospital, Aalborg, Denmark.; ^10^ Department of ORL—Head & Neck Surgery, and Copenhagen University Hospital, Copenhagen, Denmark.; ^11^ Department of Oncology, Herlev Hospital, Herlev, Denmark.; ^12^ Odense Patient data Explorative Network (OPEN), Odense University Hospital, Odense, Denmark.; ^13^ Department of Molecular Medicine, Aarhus University Hospital, Aarhus, Denmark.; ^14^ Center for Genomic Medicine, Copenhagen University Hospital, Copenhagen, Denmark.; ^15^ Department of Clinical Genetics, Odense University Hospital, Odense, Denmark.

**Keywords:** medullary thyroid carcinoma, survival, biochemical cure, nationwide, population-based, Denmark

## Abstract

***Background:*** Survival of medullary thyroid carcinoma (MTC) subgroups in relation to the general population is poorly described. Data on the factors predicting long-term biochemical cure in MTC patients are nonexistent at a population level. A nationwide retrospective cohort study of MTC in Denmark from 1997 to 2014 was conducted, aiming to detect subgroups with survival similar to that of the general population and to identify prognostic factors for disease-specific survival and long-term biochemical cure.

***Methods:*** The study included 220 patients identified from the nationwide Danish MTC cohort between 1997 and 2014. As a representative sample of the general population, a reference population matched 50:1 to the MTC cohort was used.

***Results:*** Patients diagnosed with hereditary MTC by screening (hazard ratio [HR] = 1.5 [confidence interval (CI) 0.5–4.3]), patients without regional metastases (HR = 1.4 [CI 0.9–2.3]), and patients with stage I (HR = 1.3 [CI 0.6–3.1]), stage II (HR = 1.1 [CI 0.6–2.3]), and III (HR = 1.3 [CI 0.4–4.2]) disease had an overall survival similar to the reference population. On multivariate analysis, the presence of distant metastases (HR = 12.3 [CI 6.0–25.0]) predicted worse disease-specific survival, while the absence of regional lymph node metastases (odds ratio = 40.1 [CI 12.0–133.7]) was the only independent prognostic factor for long-term biochemical cure.

***Conclusions:*** Patients with hereditary MTC diagnosed by screening, patients without regional metastases, and patients with stages I, II, and III disease may have similar survival as the general population. The presence of distant metastases predicted worse disease-specific survival, while the absence of regional metastases predicted long-term biochemical cure.

## Introduction

Medullary thyroid carcinoma (MTC) is a rare neuroendocrine tumor with an incidence of 0.19/100,000 per year and a prevalence of 3.8/100,000 inhabitants. It is divided into sporadic MTC (SMTC) and hereditary MTC (HMTC), accounting for approximately 75% and 25% of cases, respectively (1).

The survival of MTC patients is in general inferior to that of the general population. This has been demonstrated in several population-based studies (2–6). At the subgroup level, however, survival with regards to the general population is poorly described. Thus, only one study has investigated this particular aspect (6). That study concluded that survival did not differ from that of the general population in the following groups: patients with a family history of MTC detected by screening, those with a tumor size <1 cm, and those with early-stage disease at diagnosis. However, as only 60% (148/247) of the study cohort received the currently recommended thyroid surgery (total thyroidectomy) (7), it is questionable if results are applicable to contemporary MTC series.

Disease-specific survival of MTC patients is strongly dependent on the age and stage at diagnosis. This has been well recognized for decades (8–13). The significance of sex as a prognostic factor, however, remains controversial (8,10–13).

The best possible outcome for MTC patients following treatment is biochemical cure. Although being an extremely pertinent outcome, there is a paucity of data from population-based studies. In fact, only one study has investigated biochemical cure and associated predictors at a population level (12). The study, however, defined biochemical cure as normal calcitonin levels within the first six months following surgery, and therefore did not take into account the 5–21% of MTC patients who postoperatively achieve initial biochemical cure but later develop biochemical recurrence (12,14–22). Also, the study included only 36% (899/2490) of the entire MTC cohort, causing external validity issues.

Consequently, the first population-based study of an unselected nationwide MTC cohort was conducted, aiming to identify prognostic factors for long-term biochemical cure and to detect prognostic factors for disease-specific survival and MTC subgroups with survival inferior or similar to that of the general population.

## Methods

### Patients

This retrospective cohort study included 220 unique patients diagnosed with MTC in Denmark between January 1, 1997, and December 31, 2014.

An MTC cohort, initially comprising 476 patients diagnosed with MTC in Denmark between January 1960 and December 2014, was constructed through three nationwide registries: the Danish Thyroid Cancer Database, the Danish Cancer Registry, and the Danish Pathology Register (23–25). This has been described in detail previously (1,26). From this cohort, the 224 patients diagnosed nationwide in the period 1997–2014, where coverage of the entire country was considered complete, were extracted. After exclusion of four patients diagnosed at autopsy, 220 patients were included. Of these, 219 had histologically diagnosed MTC, while one had cytologically and biochemically diagnosed MTC verified by positive calcitonin staining and basal serum calcitonin >2000 pg/mL.

The investigation was approved by the Danish Health Authority (3-3013-395/3) and the Danish Data Protection Agency (18/17801).

### Methods

Data were provided by the Danish Thyroid Cancer Database. Where this was insufficient, data were drawn from the Danish Cancer Registry, the Danish Pathology Register, or medical records.

Predictor variables were sex, age, MTC type (SMTC or HMTC by screening or symptoms), tumor-node-metastasis (TNM) status, multifocality, and bilaterality. Classification of MTC type was primarily based on the absence or presence of *RE*arranged during *T*ransfection (*RET*) germline mutations. *RET* testing and classification of MTC type have been described elsewhere (1,27,28). TNM staging was performed according to the seventh and eighth editions of the American Joint Committee on Cancer Staging Manual and was based on clinical and pathological assessment (29,30). In case of discrepancy, pathological overruled clinical staging. Multifocality was defined as more than one MTC focus, while bilaterality was defined as the presence of at least one MTC focus in both lobes.

Outcome variables were overall survival, disease-specific survival, and long-term biochemical cure.

### Survival

Survival time was calculated as the time from MTC diagnosis until death, emigration, or last follow-up (January 1, 2018), whichever came first. For calculation of overall survival and disease-specific survival, all deaths and deaths due to MTC were considered as an event, respectively.

To compare overall survival between MTC patients and the general population, a reference population was created. Fifty people per each MTC patient were randomly selected from the Civil Registration System (www.cpr.dk) as controls. These were matched to the MTC case by sex, birth year, and birth month. All controls had to be alive at the date of MTC diagnosis for their respective cases. Additionally, the reference population could not include patients from the Danish MTC cohort from 1960 to 2014 (1), nor could it include first- or second-degree relatives to this cohort.

### Biochemical cure

Long-term biochemical cure was defined as undetectable basal serum calcitonin at last biochemical follow-up in patients who had received no other treatment beyond initial surgery.

### Statistical analysis

Continuous variables were reported as the median and interquartile range (IQR). Survival data were analyzed by the Kaplan–Meier method. Cox's proportional hazards regression model was employed in univariate and multivariate analyses of survival. Cox's proportional hazards models were tested for satisfying the proportional hazards assumption. If deviations from the assumption were detected, robust standard errors in the Cox model were estimated to take into account the added uncertainty. Logistic regression was used in univariate and multivariate analyses for long-term biochemical cure. *p*-Values <0.05 were considered significant. Multiple testing was adjusted by the Bonferroni method (31). All analyses were done using Stata v15.1 (StataCorp).

## Results

A total of 220 patients were included in the study. Patient characteristics are shown in Table 1. The overall female-to-male ratio was 1.47 [confidence interval (CI) 1.08–1.87].

**Table 1.** Characteristics of 220 Patients with Medullary Thyroid Carcinoma in Denmark, 1997–2014

			*Hereditary*	
	*All*	*Sporadic*	*By symptoms*	*By screening*
*Characteristics*	*(*n* = 220)*	*(*n* = 167)*	*(*n* = 10)*	*(*n* = 43)*
At diagnosis				
Sex, *n* (%)				
Female	131 (60)	105 (63)	7 (70)	19 (44)
Male	89 (40)	62 (37)	3 (30)	24 (56)
Age (years), median (IQR)	53 (39–66)	57 (45–69)	36 (18–59)	37 (22–48)
MEN2 syndrome, *n* (%)				
MEN2A	46 (87)		7 (70)	39 (91)
MEN2B	7 (13)		3 (30)	4(9)
Thyroid surgery, *n* (%)				
No thyroid surgery	12 (5)	11 (7)	0	1 (2)
Diagnostic open biopsy	5 (2)	5 (3)	0	0
Hemithyroidectomy	3 (1)	3 (2)	0	0
Total thyroidectomy	200 (91)	148 (89)	10 (100)	42 (98)
Lymph node surgery, *n* (%)				
No lymph node surgery	39 (18)	28 (17)	2 (20)	9 (21)
Lymph node extirpation	39 (18)	31 (19)	0	8 (19)
Modified neck dissection	125 (57)	93 (56)	6 (60)	26 (60)
Classic neck dissection	17 (8)	15 (9)	2 (20)	0
T category, *n* (%)				
T0	1 (0)	1 (1)	0	0
T1	91 (41)	49 (29)	5 (50)	37 (86)
T2	57 (26)	53 (32)	1 (10)	3 (7)
T3	27 (12)	23 (14)	2 (20)	2 (5)
T4	42 (19)	39 (23)	2 (20)	1 (2)
Tx	2 (1)	2 (1)	0	0
N category, *n* (%)				
N0	103 (47)	75 (45)	2 (20)	26 (60)
N1	117 (53)	92 (55)	8 (80)	17 (40)
M category, *n* (%)				
M0	199 (90)	146 (87)	10 (100)	43 (100)
M1	21 (10)	21 (13)	0	0
TNM stage,^a^*n* (%)				
I	56 (25)	31 (19)	1 (10)	24 (56)
II	40 (18)	37 (22)	1 (10)	2 (5)
III	17 (8)	10 (6)	1 (10)	6 (14)
IV	106 (48)	88 (53)	7 (70)	11 (26)
Unknown	1 (0)	1 (1)	0	0

Due to rounding up, not all sums of percentages fit.

^a^ Staging was based on the American Joint Committee on Cancer seventh and eighth editions (29,30).

IQR, interquartile range; MEN2, multiple endocrine neoplasia 2; T, tumor; N, node; M, metastasis.

In the 53 HMTC patients, the following *RET* mutations were detected: *C611W* (*n* = 3), *C611Y* (*n* = 31), *C618F* (*n* = 1), *C618Y* (*n* = 3), *C620R* (*n* = 4), *C634R* (*n* = 1), *C634Y* + *Y791F* (*n* = 1), *L790F* (*n* = 1), *V804M* (*n* = 1), *A883F* (*n* = 1), and *M918T* (*n* = 6). Several of these have been reported previously (32–39).

### Survival

The median follow-up time was 6.8 years (IQR 3.4–12.7). At last follow-up, 76 patients had died. Of these, 51 had died from MTC, while 25 had died from other causes. This yielded 5-, 10-, 15-, and 20-year overall survival rates of 75% [CI 69–80], 64% [CI 56–70], 58% [CI 49–65], and 56% [CI 47–64], respectively. Corresponding numbers for disease-specific survival were 82% [CI 76–86], 75% [CI 67–80], 71% [CI 63–78], and 69% [CI 59–77], respectively.

The reference population comprised 11,000 controls (6550 females). Table 2 shows the overall survival in MTC subgroups in relation to their corresponding reference population.

**Table 2.** Overall Survival in Subgroups of Patients with Medullary Thyroid Carcinoma in Denmark 1997–2014 in Relation to their Age- and Sex-Matched Reference Population

	*MTC population*		*Reference population*		*Univariate analysis*		
*MTC subgroup*	*Total,* n	*Deaths,* n *(%)*	*Total,* n	*Deaths,* n *(%)*	*HR*	*[CI]*	p*-Value*
All	220	76 (35)	11,000	1815 (17)	2.6	[2.0–3.4]	<0.001^d^
Age at diagnosis (years)							
<21	14	1 (7)	700	3 (0.4)	17.2	[1.8–165.7]	0.014
21–40	48	7 (15)	2400	46 (2)	8.4	[3.6–19.4]	<0.001^d^
41–60	81	22 (27)	4050	384 (9)	3.7	[2.3–5.9]	<0.001^d^
>61	77	46 (60)	3850	1382 (36)	2.7	[1.9–3.9]	<0.001^d^
Sex							
Female	131	39 (30)	6550	1054 (16)	2.2	[1.6–3.1]	<0.001^d^
Male	89	37 (42)	4450	761 (17)	3.3	[2.2–4.8]	<0.001^d^
MTC type							
HMTC, by screening	43	4 (9)	2150	135 (6)	1.5	[0.5–4.3]	0.402
HMTC, by symptoms	10	3 (30)	500	57 (11)	3.4	[1.0–10.7]	0.041
SMTC	167	69 (41)	8350	1623 (19)	2.9	[2.2–3.7]	<0.001^d^
T category^a^							
T1	91	16 (18)	4550	468 (10)	1.9	[1.1–3.3]	0.017^d^
T2	57	20 (35)	2850	435 (15)	2.7	[1.7–4.2]	<0.001
T3	27	12 (44)	1350	327 (24)	2.5	[1.2–4.9]	0.009^d^
T4	42	27 (64)	2100	557 (27)	4.6	[3.1–6.8]	<0.001
N category							
N0	103	19 (18)	5150	717 (14)	1.4	[0.9–2.3]	0.160^d^
N1	117	57 (49)	5850	1098 (19)	3.9	[2.9–5.3]	<0.001^d^
M category							
M0	199	56 (28)	9950	1549 (16)	2.1	[1.6–2.8]	<0.001^d^
M1	21	20 (95)	1050	266 (25)	31.5	[19.3–51.4]	<0.001
TNM stage^b^							
I	56	6 (11)	2800	232 (8)	1.3	[0.6–3.1]	0.525^d^
II	40	8 (20)	2000	369 (18)	1.1	[0.6–2.3]	0.696
III	17	3 (18)	850	114 (13)	1.3	[0.4–4.2]	0.617
IV	106	59 (56)	5300	1098 (21)	4.4	[3.3–6.1]	<0.001^d^
Multifocality^c^							
Yes	68	17 (25)	3400	339 (10)	2.9	[1.7–4.9]	<0.001^d^
No	132	39 (30)	6600	1012 (15)	2.3	[1.6–3.1]	<0.001
Bilaterality^c^							
Yes	51	11 (22)	2550	215 (8)	2.9	[1.5–5.6]	0.001^d^
No	149	45 (30)	7450	1136 (15)	2.3	[1.7–3.2]	<0.001

^a^ Based on 217 MTC patients with pertinent data and their corresponding reference population.

^b^ Based on 219 MTC patients with pertinent data and their corresponding reference population. Staging was based on the American Joint Committee on Cancer seventh and eighth editions (28,29).

^c^ Based on 200 MTC patients, who underwent total thyroidectomy and their corresponding reference population.

^d^ Robust standard errors were used in the Cox model to take into account the added uncertainty provided by deviations from the proportional hazards assumption.

MTC, medullary thyroid carcinoma; HMTC, hereditary MTC; SMTC, sporadic MTC; HR, hazard ratio; CI, confidence interval.

Analyses of prognostic factors for disease-specific survival in univariate and multivariate analyses are shown in Table 3. Several factors were significant on multivariate analysis (sex, and T4, N, and M category). However, when modified by Bonferroni correction, only the M category remained significant (*p* < 0.001). Conducting a similar multivariate analysis, where age at diagnosis was treated as a continuous variable, younger age significantly predicted better disease-specific survival, even after Bonferroni testing (*p* < 0.001).

**Table 3.** Disease-Specific Survival and Prognostic Factors in Patients with Medullary Thyroid Carcinoma in Denmark, 1997–2014

	*Disease-specific survival, % (*n* = 220)*				*Univariate (*n* = 220)*			*Multivariate (*n* = 217)*		
*Factors*	*5-year*	*10-year*	*15-year*	*20-year*	*HR*	*[CI]*	p*-Value*	*HR*	*[CI]*	p*-Value*
Sex										
Female	87	83	83	79	1			1		
Male	73	61	54	54	2.6	[1.5–4.5]	0.001	2.4	[1.3–4.4)	0.005
Age at diagnosis (years)										
<21	93	93	93		1			1		
21–40	93	87	87	87	1.6	[0.2–13.3]	0.686	0.6	[0.1–7.1]	0.711
41–60	85	80	71	64	3.5	[0.5–26.3]	0.224	1.1	[0.1–11.0]	0.963
>60	67	54	54		8.7	[1.2–64.5]	0.034	2.8	[0.3–29.4]	0.380
MTC type										
HMTC, by screening	100	96	96	96	1			1		
HMTC, by symptoms	77	77	77		8.8	[0.8–97.1]	0.076	5.3	[0.5–61.3]	0.182
SMTC	77	69	64		15.9	[2.2–115.5]	0.006	4.9	[0.6–42.9]	0.148
T category^a^										
T1	93	91	91	91	1			1		
T2	86	78	68	58	3.3	[1.3–8.2]	0.009	2.1	[0.8–5.5]	0.129
T3	71	64	64		4.7	[1.7–12.9]	0.003	2.3	[0.8–6.7]	0.115
T4	57	43	43		10.4	[4.4–24.4]	<0.001	2.9	[1.2–7.2]	0.022
N category										
N0	95	93	91	87	1			1		
N1	70	58	54	54	6.1	[2.9–13.0]	<0.001	2.7	[1.1–6.4]	0.025
M category										
M0	89	82	79	76	1			1		
M1	14				20.4	[10.8–38.5]	<0.001	12.3	[6.0–25.0]	<0.001^d^
TNM stage^b^										
I	98	98	98	98	1					
II	97	93	87	78	5.4	[0.6–48.3]	0.132			
III	94	87	87	87	6.9	[0.6–76.6]	0.114			
IV	64	53	49		31.8	[4.4–231.4]	0.001			
Multifocality^c^										
No	91	82	77		1					
Yes	83	78	78	78	1.2	[0.6–2.4]	0.636			
Bilaterality^c^										
Yes	86	79	79	79	1					
No	89	81	76		1.1	[0.5–2.3]	0.889			

^a^ Based on 217 patients with pertinent data.

^b^ Based on 219 patients with pertinent data. Staging was based on the American Joint Committee on Cancer seventh and eighth editions (29,30).

^c^ Based on 200 patients, who underwent total thyroidectomy.

^d^ Significant after Bonferroni correction (31).

### Biochemical cure

For assessment of long-term biochemical cure, only the 200 patients treated with at least total thyroidectomy were considered. Of these, data were available for 194 patients. Seventy (36%) and 124 (64%) patients were classified with or without long-term biochemical cure, respectively. In the latter group, five patients had undetectable calcitonin at last biochemical follow-up, but as they had received additional treatment (reoperation or external beam radiotherapy) between initial surgery and last biochemical follow up, they were regarded as not cured. Median time to last biochemical follow-up in all 194 patients was 5.5 years (IQR 3.1–9.8 years), and in the 70 patients achieving long-term cure, it was 5.6 years (IQR 3.3–10.7 years). Among the long-term cured patients, all but one (who died of other causes seven months after initial surgery) had more than two years of biochemical follow-up.

Characteristics of the patients who were long-term cured and those who were not are depicted in Table 4. Five percent (5/100) of the node-positive patients achieved long-term biochemical cure. Of these, four had only one lymph node metastasis, while one had three. Meanwhile, 69% (65/94) of the node-negative patients were cured. Analyses of prognostic factors for long-term biochemical cure in univariate and multivariate analyses are shown in Table 5.

**Table 4.** Characteristics of 194 Patients Evaluated for Long-Term Biochemical Cure^a^ Following Diagnosis of Medullary Thyroid Carcinoma in Denmark, 1997–2014

*Characteristics*	*Cured*	*Not cured*
	*(*n* = 70)*	*(*n* = 124)*
At diagnosis		
Sex, *n* (%)		
Female	49 (70)	69 (56)
Male	21 (30)	55 (44)
Age (years), median (IQR)	46 (35–57)	54 (40–66)
MEN2 syndrome, *n* (%)		
MEN2A	17 (89)	27 (84)
MEN2B	2 (11)	5 (16)
MTC type, *n* (%)		
HMTC, by screening	18 (26)	23 (19)
HMTC, by symptoms	1 (1)	9 (7)
SMTC	51 (73)	92 (74)
Thyroid surgery, *n* (%)		
Total thyroidectomy	70 (100)	124 (100)
Lymph node surgery, *n* (%)		
No lymph node surgery	15 (21)	12 (10)
Lymph node extirpation	17 (24)	14 (11)
Modified neck dissection	38 (54)	81 (65)
Classic neck dissection	0	17 (14)
T category, *n* (%)		
T0	0	1 (1)
T1	45 (64)	40 (32)
T2	16 (23)	35 (28)
T3	7 (10)	17 (14)
T4	1 (1)	31 (25)
Tx	1 (1)	0
N category, *n* (%)		
N0	65 (93)	29 (23)
N1	5 (7)	95 (77)
M category, *n* (%)		
M0	70 (100)	116 (94)
M1	0	8 (6)
TNM stage,^b^*n* (%)		
I	43 (61)	10 (8)
II	20 (29)	18 (15)
III	3 (4)	13 (10)
IV	3 (4)	83 (67)
Unknown	1 (1)	0

Due to rounding up, not all sums of percentages fit.

^a^ Long-term biochemical cure was defined as undetectable basal serum calcitonin at last biochemical follow-up in patients who had received no other treatment besides initial surgery.

^b^ Staging was based on the American Joint Committee on Cancer seventh and eighth editions (29,30).

**Table 5.** Analysis of Prognostic Factors for Long-Term Biochemical Cure^a^ in Patients who had Undergone Total Thyroidectomy for Medullary Thyroid Carcinoma in Denmark, 1997–2014

	*Univariate (*n* = 194)*			*Multivariate (*n* = 192)*		
*Factors*	*OR*	*[CI]*	p*-Value*	*OR*	*[CI]*	p*-Value*
Sex						
Female	1.9	[0.999–3.5]	0.051	1.1	[0.4–2.9]	0.867
Male	1			1		
Age at diagnosis (years)						
<21	1.7	[0.5–6.1]	0.381	5.8	[0.6–53.1]	0.120
21–40	2.9	[1.3–6.6]	0.013	2.5	[0.6–10.3]	0.197
41–60	1.9	[0.9–4.1]	0.087	1.1	[0.4–3.3]	0.844
>60	1			1		
MTC type						
HMTC, by screening	7.0	[0.8–60.8]	0.076	2.1	[0.1–38.0]	0.696
HMTC, by symptoms	1			1		
SMTC	5.0	[0.6–40.5]	0.133	3.4	[0.1–86.6]	0.454
T category^b^						
T1	34.9	[4.6–267.2]	0.001	11.3	[0.96–131.6]	0.054
T2	14.2	[1.8–113.1]	0.012	2.4	[0.2–27.0]	0.479
T3	12.8	[1.4–112.6]	0.022	4.0	[0.3–54.1]	0.291
T4	1			1		
N category						
N0	42.6	[15.7–115.8]	<0.001	40.1	[12.0–133.7]	<0.001^e^
N1	1			1		
M category^c^						
M0						
M1						
TNM stage^d^						
I	118.97	[31.1–455.1]	<0.001			
II	30.7	[8.2–114.6]	<0.001			
III	6.4	[1.2–35.1]	0.033			
IV	1					
Multifocality						
No	3.7	[1.8–7.6]	<0.001	5.0	[1.2–20.1]	0.024
Yes	1					
Bilaterality						
Yes	1					
No	1.8	[0.9–3.7]	0.110			

^a^ Long-term biochemical cure was defined as undetectable basal serum calcitonin at last biochemical follow-up in patients who had received no other treatment besides initial surgery.

^b^ Based on 192 patients with pertinent data.

^c^ Analyses could not be performed as no patients with distant metastases were biochemically cured.

^d^ Based on 193 patients with pertinent data. Staging was based on the American Joint Committee on Cancer 7th and 8th edition (29,30).

^e^ Significant after Bonferroni correction (31).

OR, odds ratio.

For the 124 patients who did not achieve long-term cure, the 5-, 10-, 15-, and 20-year disease-specific survival rates were 84% [CI 76–89], 72% [CI 61–80], 67% [CI 54–76], and 63% [CI 49–74], respectively.

## Discussion

This nationwide study reports for the first time at a population level that absence of regional lymph node metastasis is a significant predictor for long-term biochemical cure. The results also indicate that patients diagnosed with HMTC by screening, patients without regional metastases, and patients with stages I, II, and III disease may have an overall survival similar to that of the general population.

### Limitations

For optimal assessment of overall survival between the MTC subgroups and the reference population, it would have been preferable for both populations to be have been followed until all patients had died in one of the cohorts. Such follow-up, potentially spanning half a century or more, was impossible in this study, since the first patient included was from 1997. However, complete follow-up of both cohorts has been provided until January 1, 2018.

Due to the founder effect of the *C611Y* mutation in Denmark, a large proportion of the Danish multiple endocrine neoplasia type 2A (MEN2A) cohort comprise patients with *RET* mutations classified in the American Thyroid Association moderate risk level (7,39,40). Other MEN2A cohorts contain large proportions of carriers with mutations (codon 634) classified in the high risk level (27,41). Thus, assuming that MEN2A cohorts in general mirror the HMTC cohorts, the HMTC cohort in this study may not be representative of other HMTC cohorts. Therefore, it could be argued that the HMTC results predominantly apply for HMTC populations carrying moderate risk mutations, a fact that should be taken into consideration when interpreting the results. On the other hand, the distribution of carriers with moderate risk versus high risk mutations may be of lesser importance than previously thought due to recent evidence suggesting similarly aggressive clinical courses among patients with mutations from either risk level (42).

In the analysis of predictors for disease-specific survival, a larger sample size or longer follow-up may have provided more events and thus greater statistical strength. Consequently, predictors dismissed as significant after Bonferroni correction may have proven significant in an optimal setting.

It also cannot be ruled out that a small proportion in the cohort with long-term biochemical cure may develop biochemical recurrence eventually. However, with a biochemical follow-up of at least two years and no treatment other than initial surgery in 99% (69/70) of the patients, this proportion is likely very low.

### Characteristics

The MTC cohort is comparable to other large population-based MTC cohorts with regard to female-to-male ratio (6,9,43,44), age at diagnosis (6,9,43,44), distribution of MTC type (6,11,13), rates of disease-specific survival (11,12,45,46), and rates of biochemical cure (12). Concerning TNM stage, the cohort seems to differ from others (43), having a higher proportion of stage IV patients, presumably due to a high proportion of T4 and N1b (82% of all N1) patients. Also, the composition of the HMTC cohort likely differs from others due to the Danish *RET^C611Y^* founder effect causing an unusual high incidence of *C611Y* carriers in Denmark (39,40).

Notably, the age of HMTC patients diagnosed by screening did not differ compared to that of HMTC patients diagnosed by symptoms. Furthermore, 40% of HMTC patients diagnosed by screening had regional lymph node metastases. Altogether, this suggests that the diagnosis of HMTC by screening was made relatively late. In fact, this was the case, as the HMTC patients diagnosed by screening primarily comprised siblings, cousins, and parents to MEN2 index patients who were diagnosed with the syndrome at a relatively late age. This relatively late age at diagnosis of HMTC in patients detected by screening allows for the development of advanced disease and is probably also the reason for the rather low rate of long-term biochemical cure in HMTC patients diagnosed by screening.

### Survival

The Danish MTC cohort from 1997 to 2014 had a significantly worse overall survival compared to that of the general population. Not surprisingly, similar results have been demonstrated for other MTC cohorts (2–6). In the meantime, survival in HMTC patients diagnosed by screening was comparable to that of the general population. To date, only one other study has conducted a similar analysis (6). The results were equivalent to those of the current study. This study, however, strengthens the results of the other study further, as the HMTC diagnosis in the present cohort was verified by *RET* germline mutations, while the HMTC diagnosis in the other study was attached with considerable uncertainty because it was based solely on histopathological parameters, family history, and additional features of MEN2. To the best of the authors' knowledge, the present study is the first to show explicitly that patients without regional lymph node metastases have survival similar to that of the general population. This could likely be explained by the high rate of biochemical cure in this subgroup. It was also found that patients with stages I, II, and III disease had similar survival as the general population. This could indicate a negative impact on overall survival of T4 tumors and N1b and M1 disease, as these are absent in stages I, II, and III (47). Comparison to other studies is complicated by the use of different staging systems, thus hindering reasonable conclusions (2–4,6).

In univariate analysis, SMTC predicted poorer disease-specific survival (hazard ratio = 6.5 [CI 2.0–21.0]) compared to HMTC. The difference, however, disappeared in multivariate analysis. This is supported by a number of studies also finding no difference in survival between SMTC and HMTC patients after adjustment for age and stage at diagnosis (12,13,48–50).

On multivariate analysis, it was found that the presence of distant metastases significantly predicts worse disease-specific survival. This is in accordance with other recent population-based studies (9,10). As in other studies (9,10), the presence of regional metastatic disease in the Danish cohort was a weaker prognostic indicator (*p* = 0.025). Another weak predictor of adverse outcome was male sex (*p* = 0.005). Contradictory results have been found in other studies (8,10,12). To the best of the authors' knowledge, only one research group has previously reported male sex as a significant predictor (*p* = 0.0001) for disease-specific mortality at a population level (11). Our result, however, could not replicate this after Bonferroni correction. Accordingly, the influence of sex still seems unclear.

By incorporating age as a continuous rather than categorical variable in the multivariate analysis, younger age at diagnosis significantly predicted better disease-specific survival, even after Bonferroni correction. Younger age, however, may be conceived as a surrogate of early diagnosis in HMTC patients and perhaps also in SMTC patients. Recently, a large German study of 600 SMTC patients demonstrated a significant increase in the rate of MTC microcarcinomas and biochemical cure paralleled by significant declines in the proportion of node-positive patients and patients with distant metastases from 1995 to 2015 (51). These data support a time trend toward earlier detection of SMTC in Germany. The authors suggested that the trends possibly reflect greater use of calcitonin screening in patients with nodular disease on top of ultrasonography of the neck. Conducting corresponding analyses on both the SMTC and overall MTC cohort failed to identify similar significant time trends (data not shown). This may in part be due to the limited sample size, but another likely explanation is that calcitonin screening in patients with nodular thyroid disease in Denmark is not routinely used. As such, implementation of routine calcitonin screening in Denmark may potentially enhance biochemical cure rates in SMTC patients to the level reported in the German study.

### Biochemical cure

Prognostic factors for long-term biochemical cure were analyzed for the first time in an unselected cohort at a population level. On multivariate analysis after Bonferroni adjustment, regional lymph node metastasis was found to be the only significant predictor (*p* < 0.001). Only one other population-based study has investigated prognostic factors for biochemical cure (12). Their results showed that stage at surgery was the only significant indicator for biochemical cure initially postoperatively. However, the study was limited by an inclusion of only 36% of all potential MTC patients and did not elaborate on the stage subgroups. When considering institutional series investigating the association between nodal metastasis and biochemical cure by multivariate analysis, the present results are in keeping with most (18,52–54), but not all studies (55). Disagreement with the latter study may be explained by a difference in study cohorts, as the latter study only included patients with tumor size <1.6 cm. Of note, five patients in the present cohort with node-positive MTC achieved long-term biochemical cure. In these patients, the number of metastatic lymph nodes did not exceed four. Conversely, no patients with more than four positive lymph nodes were cured. Corresponding trends have been demonstrated in institutional series (52,53,56,57). Two of these found that initial postoperative biochemical cure was virtually impossible in patients with >10 lymph node metastases but could be obtained in 31% (8/26) and 57% (17/30) of patients with <10 lymph node metastases (56,57). One study, also investigating initial postoperative biochemical cure, found a mean of 1.5 and 12.0 positive lymph nodes in cured and not cured patients, respectively (53). Another study, reporting on long-term biochemical cure, found a mean of 2.4 and 10.1 metastatic lymph nodes in cured and not cured patients, respectively (52). Combined with the results from the present nationwide study, this lends hope that long-term biochemical cure may be possible in MTC patients with regional lymph node metastases. However, the number of positive nodes has to be very low.

## Conclusions

Patients with hereditary MTC diagnosed by screening, patients without regional metastases, and patients with stages I, II, and III disease may have similar survival as the general population. The presence of distant metastases predicted worse disease-specific survival, while the absence of regional metastasis predicted long-term biochemical cure.
